# Gut microbial shifts by synbiotic combination of *Pediococcus acidilactici* and lactulose in weaned piglets challenged with Shiga toxin-producing *Escherichia coli*

**DOI:** 10.3389/fvets.2022.1101869

**Published:** 2023-01-12

**Authors:** Robin B. Guevarra, Eun Sol Kim, Jin Ho Cho, Minho Song, Jae Hyoung Cho, Jun Hyung Lee, Hyeri Kim, Sheena Kim, Gi Beom Keum, Chan Ho Lee, Won Tak Cho, Suphot Watthanaphansak, Hyeun Bum Kim

**Affiliations:** ^1^Department of Animal Resources Science, Dankook University, Cheonan, Republic of Korea; ^2^Division of Food and Animal Sciences, Chungbuk National University, Cheongju, Republic of Korea; ^3^Division of Animal and Dairy Science, Chungnam National University, Daejeon, Republic of Korea; ^4^Gene Bio Tech Co., Ltd., Gongju, Republic of Korea; ^5^Department of Veterinary Medicine, Faculty of Veterinary Science, Chulalongkorn University, Bangkok, Thailand

**Keywords:** microbiota, piglet, Shiga-toxin producing *Escherichia coli*, 16S rRNA gene, synbiotics

## Abstract

Development of alternatives to in-feed antibiotics in the swine industry have been the focused of many pig gut microbiota studies to improve animal health. In this study, we evaluated the effects of probiotic *Pediococcus acidilactici* (PRO), prebiotic lactulose (PRE), and their synbiotic combination (SYN) on gut microbiota using 16S rRNA gene sequencing in weaned piglets challenged with Shiga-toxin producing *Escherichia coli* (STEC). Our data showed that prebiotics, probiotics and synbiotics improved the intestinal health in weaned piglets. No significant differences were observed in species richness and species diversity in weaned piglets fed prebiotics, probiotics and their synbiotic combination. However, beta diversity analysis revealed distinct clustering of the microbiota of according to dietary treatment and by oral challenge of STEC. At the phylum level, Firmicutes to Bacteroidetes ratio was lower in the dietary treatment groups than the control group. Oral supplementation of prebiotics, probiotics and synbiotics enriched the abundance of *Prevotella* and *Roseburia*. *Succinivibrio* was elevated in PRO group; however, *Phascolarctobacterium* was depleted with STEC challenge regardless of dietary treatment. Overall, our data showed that administration of synbiotics in piglets improved intestinal health through gut microbiota modulation. Our data indicated that prebiotics, probiotics and their synbiotic combination could promote intestinal health through gut microbiota modulation in weaned piglets.

## Introduction

Mortality of piglets at weaning causes major economic losses and it is a serious concern for the global pork industry (1). Weaning is a critical and stressful stage in the life cycle of pigs and it is frequently associated with severe enteric infections such as post-weaning diarrhea (2). Biological stresses during the first week after weaning induce changes in the intestinal barrier function and structure leading to poor growth performance such as reduced feed intake, slow growth rate and reduced feed conversion of post-weaning piglets (3). The weaning transition generally causes gastrointestinal infections which are associated with piglet mortality of ~15% in the swine industry (1). Antibiotic growth promoters, which are mainly used to treat and prevent disease and improve growth rate, have been banned in many developed countries including the United States and in the European Union due to increased conflicts about drug residues and antimicrobial resistance (4). Hence, there is a need for alternatives to in-feed antibiotics to reduce mortality and to improve gut health in pigs at the critical weaning period.

The intestinal microbiota has received a lot of attention in the recent years because of its role in immune system development and function, and for improvement of overall health, growth and performance of pigs (5). To mitigate the negative impact of the stress on early-weaned piglets, effective measures are required to promote gut health. The most widely researched antibiotics alternatives include probiotics (6), prebiotics (7) and their combination known as synbiotics. The effects of probiotics and prebiotics in the swine gut microbiota have been demonstrated in a number of studies. For example, probiotic *Lactobacillus plantarum* PFM 105, isolated from the rectum of a healthy sow, showed to improve the growth and promote intestinal development through modulation of gut microbiota in weaning piglets (8). Interestingly, the prebiotic lactulose has been shown to significantly increase the fecal diversity, decrease the abundance of pathogenic bacteria and increase the number of beneficial bacteria in weaned piglets (9). However, evaluation of synbiotics to improve gut health and improve nutrient utilization have received less much attention compared to other alternatives. Moreover, limited information is available regarding the protective effects of synbiotics on intestinal microbiota to control post-weaning diarrhea in piglets. Further research is still needed in this area since the perfect alternative to antibiotics does not yet exist and the effects of probiotics on swine gut microbiota are relatively limited and often contradictory.

Shiga toxin-producing *Escherichia coli* (STEC) is an important pathogen, which can cause pig diseases, including hemorrhagic colitis (10). The STEC bacterium is characterized by the ability to produce a cytotoxin, known as Shiga toxin (*Stx*), which is encoded by stx genes carried on bacteriophages (11). Pigs are important reservoir for human STEC infections, hence effective mitigation strategies including the use of prebiotics, probiotics, and synbiotics are required to improve animal health and address public health concerns.

In this study, we evaluated the effects of probiotic *Pediococcus acidilactici* (PRO), prebiotic lactulose (PRE), and their synbiotic combination (SYN) on weaned pig gut microbiota using 16S rRNA gene sequencing in weaned piglets challenged with Shiga-toxin producing *Escherichia coli* (STEC).

## Materials and methods

### Animals and housing

A total of 50 healthy weanling pigs [Duroc x (Landrace x Yorkshire)] with average body weight of 5.33 ± 0.60 kg weaned at the age of 28 days were used in this study at experimental research center of Dankook University, Cheonan, South Korea. All pigs in this study were selected from one delivery room and had similar husbandry practices. Each pen was equipped with a one-sided self-feeder and a nipple water-feeder for *ad libitum* access to feed and water throughout the experiment. The experimental procedures used in this study were approved by the Animal Care and Use Committee of Dankook University (No. DK-1-1645).

### Diet and experimental design

On the day of weaning, piglets were divided into five groups consisting of ten pigs per treatment and housed in pens of five animals per pen using a randomized complete block design for the 7 weeks trial. Basal diet was provided in a mash form and formulated to meet or exceed the nutrient requirements (Table 1). The 5 dietary treatments were: (i) CONT, basal diet without any antibiotics or feed supplements, (ii) PRE, basal diet + 0.05% prebiotics, (iii) PRO, basal diet + 0.1% probiotics (*Pediococcus acidilactici*), (iv) SYN1, basal diet + 0.05% synbiotics, (v) SYN2, basal diet + 0.1% synbiotics. The synbiotics was formulated with a prebiotic lactulose at a concentration of 10 g/kg feed combined with *Pediococcus acidilactici* GB-U15 KCCM 11856P at a concentration of 5.0 x 10^9^ colony forming units (CFU)/mL. The synbiotics used in the study were provided by Genebiotech Co., Ltd. (Seoul, Korea). We selected lactulose as the prebiotics, the low doses of lactulose can help to stimulate the growth of health-promoting bacteria in the gastrointestinal tract.

**Table 1:** Composition of basal diet for weaned pigs (as-fed basis).

**Item**	**Diet**
**Ingredient (%)**	
Corn	56.09
Soybean meal, 44%	26.00
Soy protein concentrate	12.00
Soybean oil	3.00
Limestone	1.30
Monocalcium phosphate	1.20
Vit-Min premix^a^	0.04
L-lysine-HCl	0.24
DL-methionine	0.09
L-threonine	0.04
Total	100
**Calculated energy and nutrient contents**	
ME, Mcal/kg	3.48
CP, %	24.17
Calcium, %	0.84
Phosphorus, %	0.66
Lysine, %	1.54
Methionine, %	0.45
Cysteine, %	0.39
Threonine, %	0.96
Tryptophan, %	0.28
Arginine, %	1.60
Histidine, %	0.67
Isoleucine, %	1.03
Leucine, %	2.05
Phenylalanine, %	1.21
Valine, %	1.09

a Provided per kilogram of diet: vitamin A, 12,000 IU; vitamin D3, 2,500 IU; vitamin E, 30 IU; vitamin K3, 3 mg; D-pantothenic acid, 15 mg; nicotinic acid, 40 mg; choline, 400 mg; and vitamin B12, 12 μg; Fe, 90 mg from iron sulfate; Cu, 8.8 mg from copper sulfate; Zn, 100 mg from zinc oxide; Mn, 54 mg from manganese oxide; I, 0.35 mg from potassium iodide; Se, 0.30 mg from sodium selenite.

### STEC challenge and clinical evaluation

At week 5 of the experiment (56-day-old), five pigs from each group were inoculated with the pathogenic STEC to determine the impact of the dietary treatments on intestinal microbiota of piglets challenged with STEC. STEC strain used in this study was isolated from the sick pig and had the virulent genes, such as F18, F6, heat-labile enterotoxin (LT), Shiga toxin type 2 (*stx2)*, Shiga toxin type 2e (*stx2e*) genes. The STEC, which was isolated from piglet feces, was grown in fresh LB broth and incubated at 37°C with shaking for 24 h. The final concentration of *E*. *coli* used was ~2 x 10^9^ CFU/mL. Five piglets from each group orally inoculated with 5 mL of *E*. *coli* (2 x 10^9^ CFU/mL) diluted in PBS. PBS was orally administered in the remaining five piglets from each group. The health status of piglets during the experiments was assessed by fecal consistency scoring using a five-grade system. The scoring system for stool consistency that indicate stool hardness or softness is as follows: 1 = hard, dry pellets in a small, hard mass; 2 = hard, formed stool that remains firm and soft; 3 = soft, formed and moist stool that retains its shape; 4 = soft, unformed stool that assumes the shape of the container; 5 = watery, liquid stool that can be poured.

### Fecal collection and intestinal histology

Fresh fecal samples were collected individually from the rectum of each piglet at week 7 of the experiment. Two pigs from each group were euthanized at week 7 of the experiment (2 weeks after *E. coli* oral challenge) for intestinal morphological analysis. The proximal segments of ileum, colon and cecum were sampled for histological examination. For histology, 3-cm sections from the ileum, cecum and colon were removed, opened longitudinally, and fixed in a 10% neutral formalin solution. Tissue samples were dehydrated and embedded in paraffin wax, sectioned at 5 μm, and stained with hematoxylin and eosin. Morphological measurements were performed with a light microscope.

### Genomic DNA extraction

Total DNA from the feces was extracted from 200 mg of feces per sample using QIAamp Fast DNA Stool Mini Kit (QIAGEN, Hilden, Germany) according to the manufacturer's instructions. Cell lysis was performed by bead-beating the samples twice for 2 min at 300 rpm, with an incubation period of 5 min in a water bath at 70°C between beatings. The concentrations of DNA were measured using a Colibri Microvolume Spectrometer (Titertek Berthold, Pforzheim, Germany) and samples with OD260/280 ratios of 1.80–2.15 were processed further.

### 16S rRNA gene library preparation and sequencing

The PCR primers 799F-mod6 (5′ CMGGATTAGATA CCCKGGT-3′) and 1114R (5′-GGGTTGC GCTCGTTGC-3′) were used to amplify the V5 to V6 hypervariable regions of the 16S rRNA genes. The amplification mix contained 5 X PrimeSTAR Buffer (Mg^2+^) (Takara Bio, Inc., Shiga, Japan), 2.5 mM concentrations of each deoxynucleotide triphosphates, 2.5 U/μL of PrimeSTAR HS DNA Polymerase, 10 pmol of each primer, and 25 ng of DNA in a reaction volume of 50 μL. The thermal cycling parameters were as follows: initial denaturation was at 98°C for 3 min, followed by 30 cycles of 98°C for 10 s, 55°C for 15 s, and 72°C for 30 s, and a final 3-min extension at 72°C. PCR products were purified using PCR a Wizard^®^ SV Gel and PCR Clean-Up System purification kit, (Promega, Wisconsin, USA).

After sample preparation and quality control, 16S rRNA gene amplicons were sequenced using the Illumina MiSeq platform at Macrogen Inc. (Seoul, Republic of Korea) according to the manufacturer's instructions. Briefly, the sequencing library was prepared by random fragmentation of the DNA samples followed by 5′ and 3′ adapter ligation. This step attaches dual indices and Illumina sequencing adapters using the Nextera XT Index Kit. The final products were normalized and pooled using the PicoGreen, and the size of libraries were verified using the TapeStation DNA ScreenTape D1000 (Agilent). The PCR conditions were as follows: initial denaturation (3 min at 95°C) 8 amplification cycles (95°C for 30s, 55°C for 30s, 72°C for 30s) and final elongation (72°C for 5 min).

### 16S rRNA gene analysis

All the raw sequence data from Illumina MiSeq platform were checked for quality using FastQC. Then, Mothur software was used to remove low-quality sequences (12). Briefly, sequences that did not match the PCR primers were eliminated from demultiplexed sequence reads. Sequences containing ambiguous base calls and sequences with a length of <200 bp were also removed to minimize the effects of random sequencing errors. Chimeric sequences were identified and excluded for downstream analysis using the UCHIME algorithm implemented in Mothur. Next, the QIIME (Quantitative Insights into Microbial Ecology) pipeline (version 1.9.1) was used to perform operational taxonomic unit (OTU) picking using the open-reference OTU picking workflow with the SortMeRNA and SUMACLUST methods for reference OTU and *de novo* OTU picking, respectively. Taxonomy was assigned using the naïve Bayesian Ribosomal Database Project (RDP) classifier based on GreenGenes taxonomy reference database version 13_8. Low-abundance OTUs and singletons were filtered from the OTU table for downstream analysis with minimum count of 4 and low-count filter based on 20% prevalence in samples. Then, data normalization was performed by rarefying the data to the minimum library size and by data scaling using the total sum scaling before any statistical comparison to address the variability in sampling depth and the sparsity of the data.

### Statistical analysis

Statistical analysis was performed using the R package MicrobiomeAnalystR and GraphPad Prism v7.00 (La Jolla, CA, USA). Alpha diversity measures including observed OTUs, Chao1, Shannon and Simpson indices were computed using the MicrobiomeAnalystR. Significant differences in alpha diversity among the groups and pairwise comparisons were calculated based on analysis of variance tests. Significant difference level was set at *P* < 0.05. The principal coordinate analysis (PCoA) plots at the OTU level based on the weighted and unweighted UniFrac distances. Significant differences in beta-diversity were performed using the Analysis of Similarities (ANOSIM) based on the unweighted and weighted UniFrac distances. The heatmap of core microbiota was performed with the default parameters at 20% sample prevalence and 0.2% relative abundance.

## Results

### Growth performance and intestinal morphology

The effects of synbiotics administration on growth performance of weaned piglets is shown in Supplementary Table 1. Final body weight, average daily gain, and gain to feed ratio were significantly increased in SYN1 and SYN2 group compared to the CONT, PRO and PRE groups, suggesting that supplementation of synbiotics may improve growth performance of weaned piglets. The effects of prebiotic, probiotic and synbiotic supplementation on fecal index is shown in Supplementary Table 2. Piglets of the SYN1 and SYN2 group significantly decreased the fecal score (*P* < 0.05), while no differences were observed between PRE and PRO as compared to the CONT group suggesting that synbiotics administration decreased the diarrhea incidence in weaned piglets.

Histological examinations of ileal tissue revealed that STEC-challenged CONT group increased the inflammatory cells including neutrophils and macrophages in the lamina propria as compared to the healthy CONT group, which were given PBS only. Interestingly, oral administration of PRE, PRO, SYN1 and SYN2 group decreased these inflammatory cells (Supplementary Figure 1). In the cecum tissue, STEC-challenged CONT group expanded the mucosal crypt and increased the number of plasma cells as compared to the healthy CONT group. However, these observations were lower in STEC-challenged pig groups fed PRO, PRE, SYN1 and SYN2 (Supplementary Figure 2). In the colon tissue, neutrophils and plasma cells were increased in the STEC-challenged CONT group compared to the healthy CONT group. Similarly, PRO, PRE, SYN1 and SYN2 decreased these inflammatory cells with higher reduction in the SYN2 (Supplementary Figure 3).

### DNA sequencing data

Total DNA was extracted from fecal samples of pigs and the extracted community DNA was PCR amplified and sequenced using primers specific for the V5 to V6 hypervariable regions of the 16S rRNA genes. The 16S rRNA gene sequencing produced a total of 4,875,951 raw sequence reads from 48 fecal samples ranging from 66,397 to 165,469 reads. The average quality score (Phred scores) across all the samples ranged from 32 to 36. Phred scores >Q30 indicated that that there was <0.1% chance that a base was called incorrectly. Further data filtering was performed in the OTU table to remove low quality sequences and to improve downstream statistical analysis (Supplementary Figure 4). The total number of reads after quality filtering was 3,409,184 ranging from 46,296 to 118,099 with an average counts per sample of 71,024. For alpha and beta diversity analyses, all samples were rarefied to the minimum number of sequences to account for unequal sequencing depth. A total of 1,688 OTUs were obtained after data filtering.

### Alpha diversity

Alpha diversity indices were compared between the treatment groups (PRE, PRO, SYN1, and SYN2) as compared to the CONT. Also, the alpha diversity between the STEC-challenged treatment groups and their non-challenged counterpart were compared to determine the effects of STEC on alpha diversity and the protective effects of PRE, PRO, SYN1, and SYN2. The rarefaction curves for 16S rRNA gene sequences of all the samples with an OTU definition at 97% identity cut-off were shown in Supplementary Figure 5, indicating that sampling depth was sufficient for downstream OTU-based analysis. The number of observed OTUs and Chao1 were used to measure species richness, whereas Shannon and Simpson diversity indices were used to measure species diversity.

**Figure 1:**
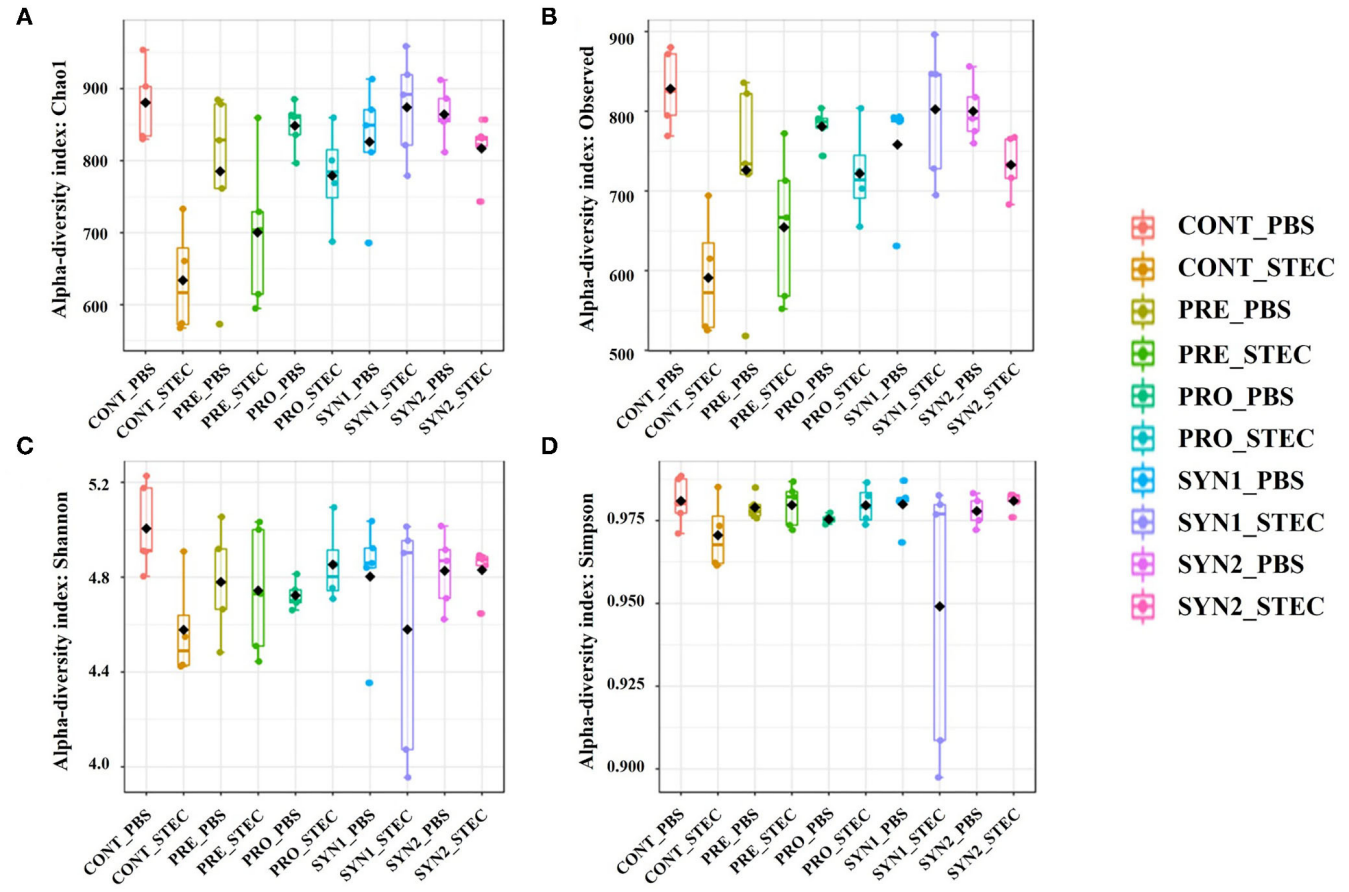
Box plots showing the alpha diversity indices of the pig groups PRE, PRO, SYN1 and SYN2 with or without STEC challenge. Species richness was measured using **(A)** Chao1 index and **(B)** number of observed OTUs, while species diversity was measured using **(C)** Shannon and **(D)** Simpson diversity indices. Boxes represent the interquartile range (IQR) between the 25th and 75th percentile, and the black dot inside the box denotes the median value. Whiskers represent the lowest and highest values within 1.5 time from the 25th and 75th quartiles, respectively. Boxes were colored according to treatment group (CONT, PRE, PRO, SYN1, SYN2) challenged with STEC or PBS.

Interestingly, Chao1 and observed OTUs were significantly lower in the CONT challenged with STEC compared to those of CONT without STEC challenge, indicating that STEC challenge significantly decreased the microbial species richness (Figures 1A, B). However, no significant differences in Chao1 and number of observed OTUs were observed between the PRE, PRO, SYN1, and SYN2 and their STEC-challenged counterparts, suggesting that probiotics, prebiotics or their combination may play a role in maintaining the balance of microbial communities in the gut of piglets against STEC infection (Figures 1A, B).

In addition, oral challenge of STEC significantly decreased the Shannon diversity in weaned piglets as compared to the non-challenged CONT group, indicating that STEC infection altered the species diversity of microbial communities (Figure 1C). Furthermore, no significant difference in Simpson diversity was observed between the treatment groups compared to the non-challenged healthy CONT group, and between those groups infected with STEC compared to the STEC-challenged CONT group (*P* > 0.05) (Figure 1D).

### Beta diversity

**Figure 2:**
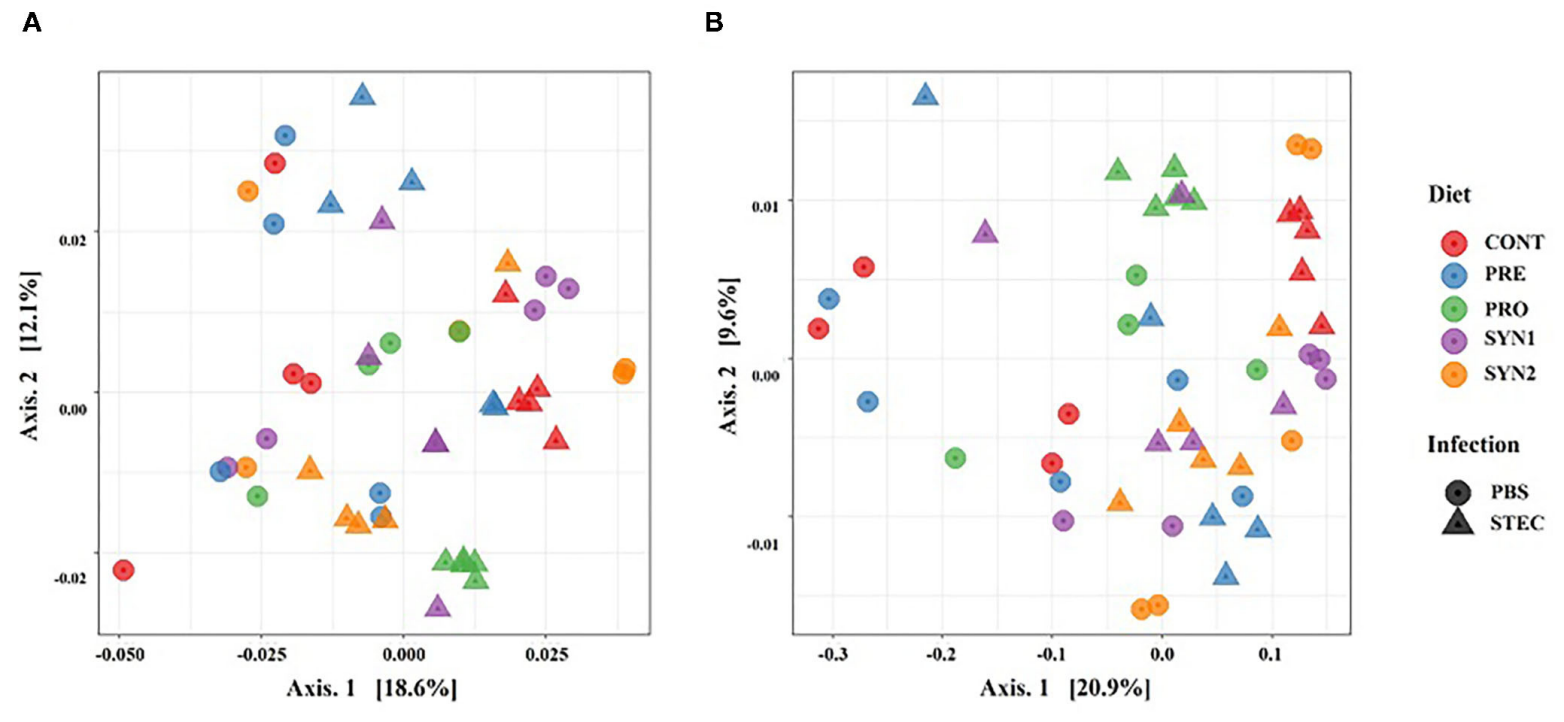
Beta diversity analysis of the pig gut microbiota of the pig groups PRE, PRO, SYN1 and SYN2 with or without STEC challenge. Principal coordinates analysis (PCoA) plots based on the weighted **(A)** and unweighted **(B)** UniFrac distances of gut microbial communities. Each symbol represents the microbiota from individual pig sample and were color coded according to treatment group (CONT, PRE, PRO, SYN1 and SYN2) while shape represents the oral challenge with STEC **(circle)** and PBS **(triangle)**. The axes show the percent variation.

Beta diversity, which is defined as the diversity among the treatment groups, was measured using the weighted and unweighted UniFrac distances with the former takes into account the relative abundance of species and the latter considers the presence or absence of OTUs in the community. Principal coordinate analysis (PCoA) was used to visualize the separation of microbial community among the treatment groups (CONT, PRE, PRO, SYN1, SYN) and effects of STEC infection. The PCoA plots based on both unweighted (Figure 2A) and weighted UniFrac (Figure 2B) distances showed significant differences in the separation of microbial communities in pigs in response to the different treatment groups (PRE, PRO, PRO, SYN1, and SYN2) and oral challenge of STEC as measured using ANOSIM (*P* < 0.05). PCoA results indicated that fecal microbial communities differ in pigs between treatment group (PRE, PRO, SYN1, and SYN2) and CONT groups. Moreover, separation of microbiota obtained were also significantly different between the unchallenged treatment groups and pigs treatment groups orally challenged with STEC based on ANOSIM (*P* < 0.05). These results suggest that prebiotics, probiotics and their synbiotic combination had individual effects on the intestinal microbial community structure in pigs.

### Microbial compositions associated with the administration of prebiotics, probiotics, and synbiotics in weaned piglets

**Figure 3:**
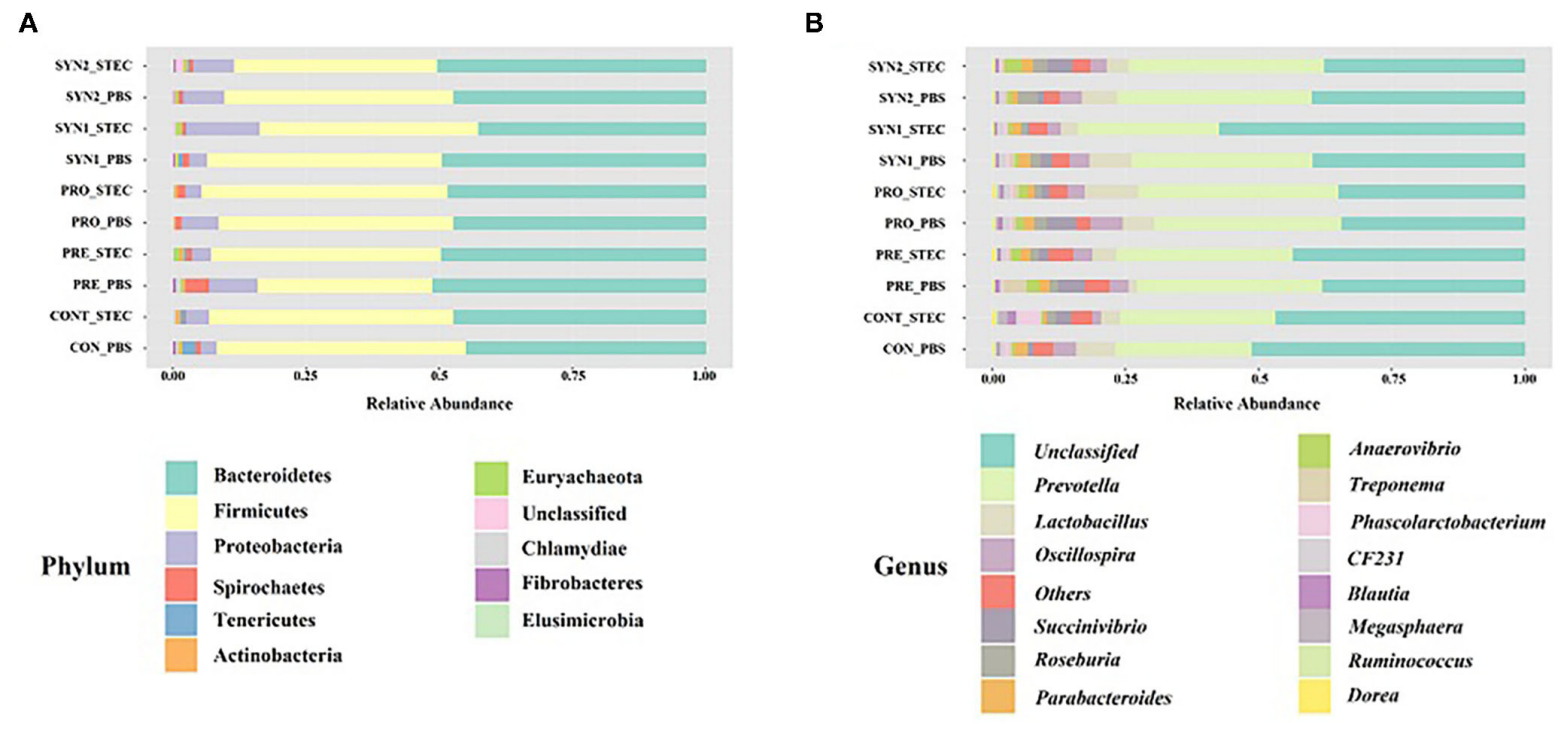
Gut microbiota composition of the pig groups PRE, PRO, SYN1 and SYN2 with or without STEC challenge. Bar plots showing, the relative abundance of taxa at the phylum **(A)** and genus **(B)** levels in the different treatment groups (CONT, PRE, PRO, SYN1 and SYN2) orally challenged with STEC or PBS.

We examined the bacterial compositions associated with oral administration of probiotics, prebiotics and synbiotics and STEC oral challenge in weaned piglets. At the phylum level, a total of 11 phyla were identified and the top 5 most abundant phyla were Bacteroidetes (42.63–51.10%), Firmicutes (32.94–46.78%), Proteobacteria (3.11–13.82%), Spirochaetes (0.19–4.48%), and Tenericutes (0.05–2.61%). Phylum Bacteroidetes and Firmicutes collectively ranged from 83.70 to 94.58% of the total sequences among the groups (Figure 3A).

At the genus level, 50 unique genera were identified from at least one sample in each group. Regardless of the treatment group, the top 5 most abundant genera were *Prevotella* (25.73–37.52%), *Lactobacillus* (1.59–9.97%), *Oscillospira* (1.60–6.24%), *Succinivibrio* (0.27–5.63%), and *Roseburia* (0.26–3.72) (Figure 3B).

**Figure 4:**
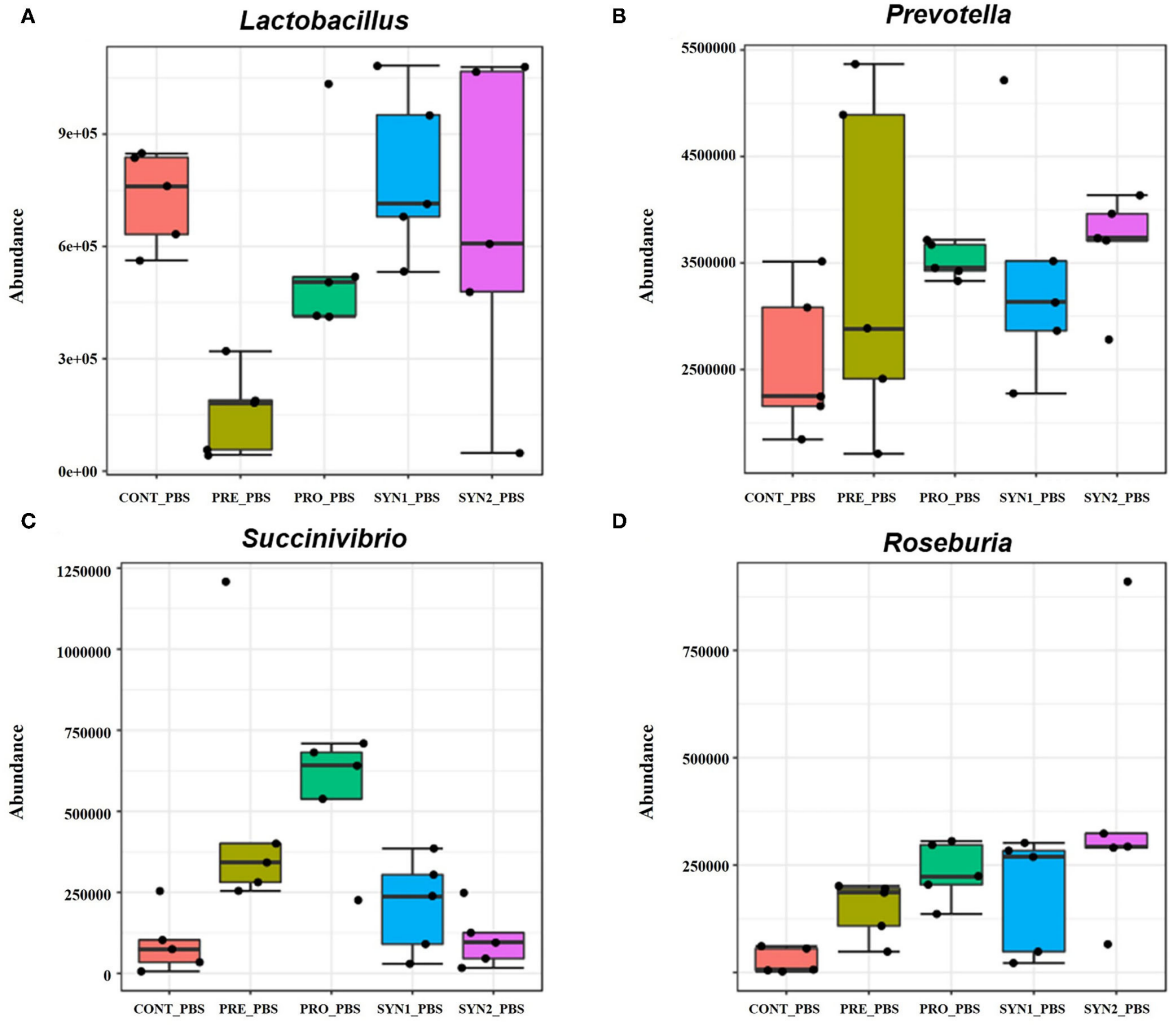
Box plots showing significantly different genera in weaned pigs supplemented with prebiotics, probiotics or synbiotics without STEC challenge. Abundance of bacterial genera including **(A)** *Lactobacillus*, **(B)** *Prevotella*, **(C)** *Succinivibrio*, and **(D)** *Roseburia* were compared between PRE, PRO, SYN1 and SYN2 in comparison to the negative CONT group.

Without STEC challenge, differential abundance analysis revealed that each additive resulted in different alterations of the pig intestinal microbiota at the genus level (Figure 4). Relative bundance of *Lactobacillus* was not different among PRO, SYN1 and SYN2 groups (Figure 4A). One of the most interesting observations of the present study is the significant increase in the abundance of *Prevotella* in all the dietary treatments (PRE, PRO, SYN1 and SYN2) as compared to the CONT (*P* < 0.05) (Figure 4B). Moreover, abundance of *Roseburia* was significantly elevated by oral administration of PRE, PRO and SYN2 as compared to the negative CONT group (*P* < 0.05) (Figure 4C). Another interesting finding of the study is the significant increase of *Succinivibrio* in the PRO group as compared to the CONT group (*P* < 0.05) (Figure 4D). These findings indicate that prebiotics, probiotics and synbiotics have unique effects on gut microbial composition in weaned piglets.

### Differential abundance in microbial composition associated with STEC infection in weaned piglets receiving prebiotics, probiotics, and synbiotics

**Figure 5:**
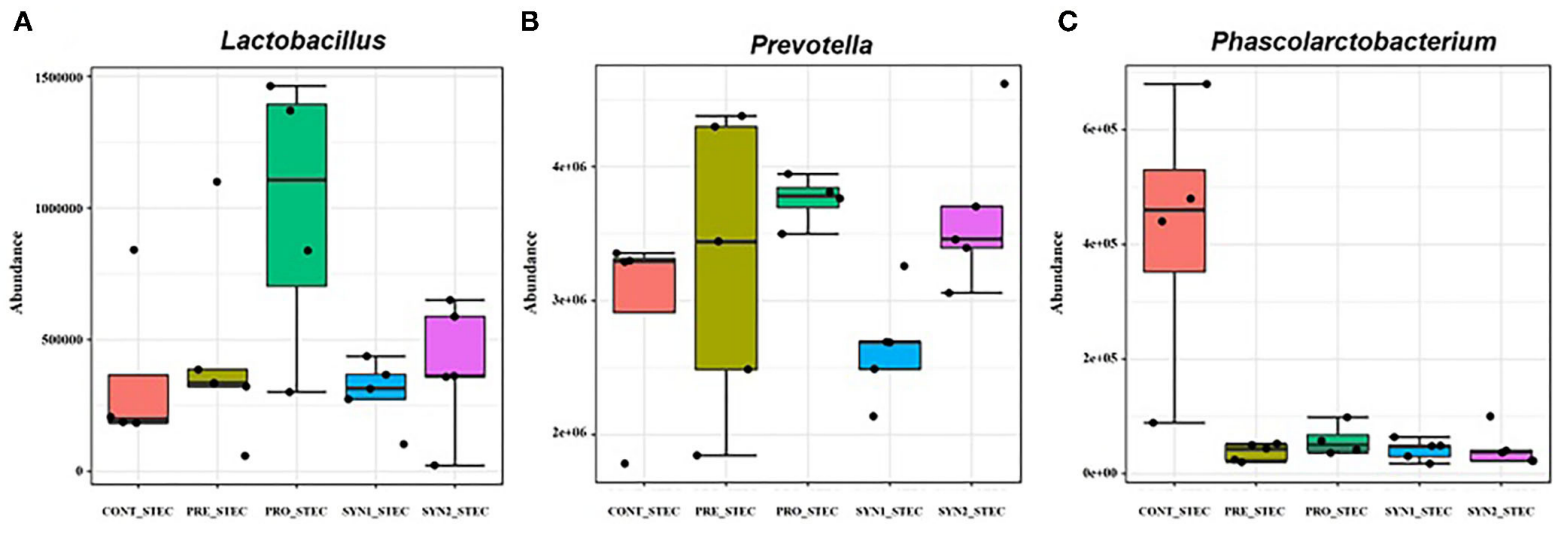
Box plots showing significantly different genera in weaned pigs supplemented with prebiotics, probiotics or synbiotics with STEC challenge. Abundance of bacterial genera including **(A)** *Lactobacillus*, **(B)** *Prevotella*, **(C)** *Phascolarctobacterium* were compared between PRE, PRO, SYN1 and SYN2 in comparison to the STEC-challenged CONT group.

Differences in microbial composition at the genus level between non-challenged and STEC-challenged CONT groups were compared to determine the effects of STEC infection in weaned piglets (Figure 5). STEC-challenge resulted to significant increase in the population of *Phascorlarctobacterium* and *Prevotella* while there was a significant decrease in abundance of *Lactobacillus* in comparison to the CONT pigs fed PBS (*P* < 0.05). No significant differences were observed between STEC-challenged CONT and PRE group challenged with STEC (*P* > 0.05). On the other hand, a significant increase in the abundance of *Prevotella* and *Lactobacillus* and significant reduction of *Phascolarctobacterium* were observed in PRO group challenged with STEC (*P* < 0.05). However, in SYN1 group challenged with STEC, significant depletion in the abundance of *Prevotella* and *Phascolarctobacterium* were observed (*P* < 0.05). In the SYN2 group challenged with STEC, we observed a significant increase in the relative abundance of *Prevotella* and a significant depletion of *Phascolarctobacterium* similar to those observations in the PRO group with STEC (*P* < 0.05). These findings suggest that STEC infection significantly altered the composition of the pig gut microbiota, however, prebiotics, probiotics or their synbiotic combination have inhibitory effects to fight against STEC.

### Core microbiota in weaned piglets orally administered with prebiotics, probiotics, and synbiotics

**Figure 6:**
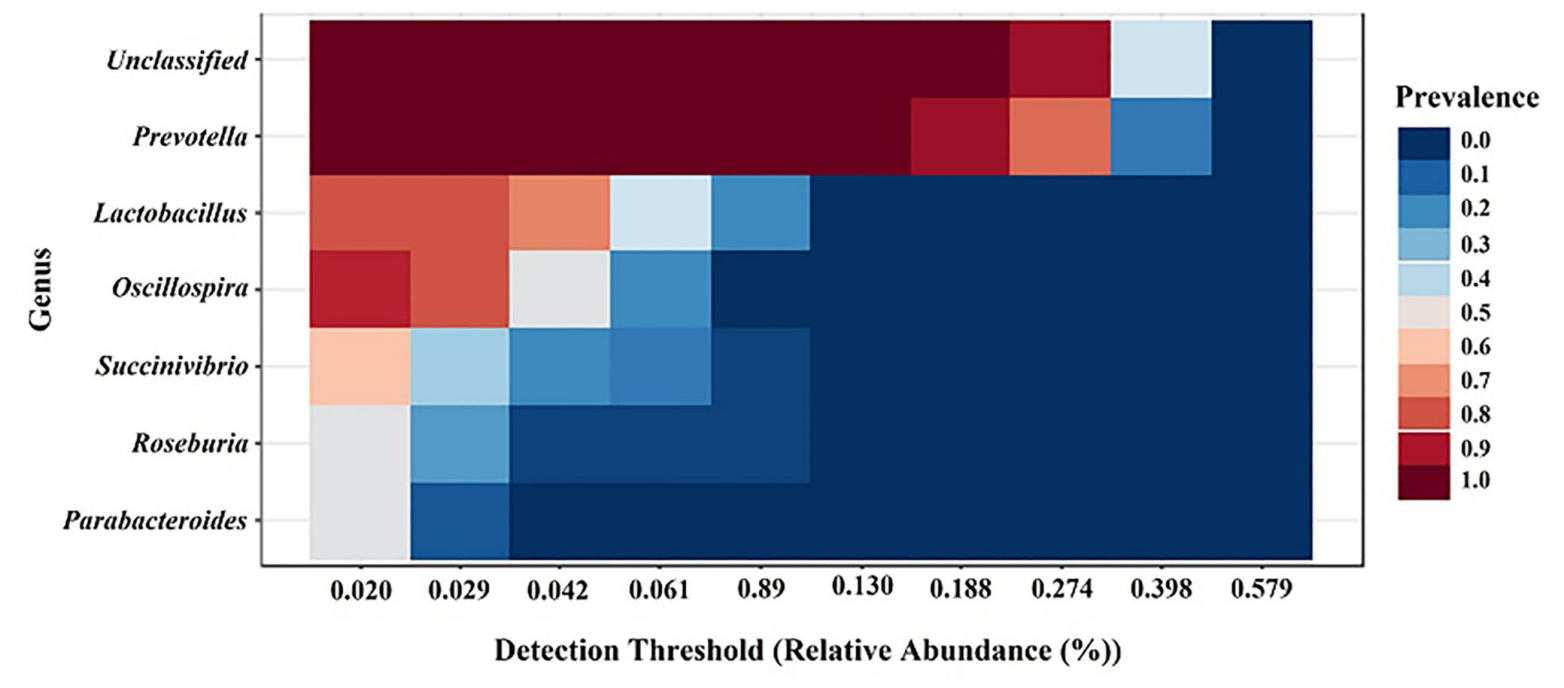
Core gut microbiota among the groups regardless of treatments. Heatmap depicting the core OTUs and their prevalence at different detection thresholds.

The core microbiome analysis was performed at the genus level based on the sample prevalence and relative abundance cut-off value at 20 and 0.02%, respectively. Regardless of the treatment group, the six core bacterial genera were identified as *Prevotella, Lactobacillus, Oscillospira, Succinivibrio, Roseburia*, and *Parabacteroides*, which were shown in descending order according to prevalence (Figure 6). Our results were similar to a previous study on meta-analysis of the swine gut microbiota using published data sets from 16S rRNA gene sequences to define a core microbiota in the pig gut (13). These findings suggest that pig gut microbiota may be used for future gut microbiota manipulation studies for potential health benefits.

## Discussion

In this study, we investigated the influence of prebiotics, probiotics and their synbiotic combination on the intestinal microbial composition in weaned pigs. Probiotics and prebiotics are two commonly used feed additives in swine nutrition and they have been extensively studied due to their perceived health benefits (14). Probiotics are defined as “live microorganisms which, when consumed in adequate amounts as part of food, confer a health benefit on the host” (5, 15). On the other hand, prebiotics are defined as “a non-digestible food ingredient that beneficially affects the host by selectively stimulating the growth and/or activity of one or a limited number of bacteria in the colon and thus improves health” (16). To date, the focus has been primarily around the use of prebiotics and probiotics for reducing post-weaning diarrhea, with little research evaluating synbiotics as possible interventions on pig gut microbiota modulation. Post-weaning diarrhea has been a particular concern to the swine industry, hence many studies including the use of prebiotics and probiotics as alternatives to in-feed antibiotics have been reported to improve swine gut health and reduce mortality in weaning piglets (17, 18). However, only a few of them have been performed with STEC challenge in weaned piglets. The present study revealed that mortality was reduced by synbiotic administration and improved gut health by reduction of inflammatory cells in the small intestine in weaned piglets.

In this study, prebiotics, probiotics and their synbiotic combination had no significant effects on species richness and species diversity in weaned piglets. There were no significant differences in the number of observed OTUs and Chao1 were detected in pigs receiving probiotics, prebiotics or their synbiotic combination, indicating that these feed additives had no effects on species richness in the fecal microbiota of pigs. These observations were similar with that of Wang et al. (8) who found that alpha diversity indices were not affected by treatment with probiotics in weaned piglets. Umu et al. (19) also revealed that prebiotic alginate and resistant starch diet decreased the species richness in the swine microbiome. Our results were also in agreement with a previous study in infant microbiome which indicated that probiotic supplementation did not alter the overall bacterial community richness and evenness (20). In contrast, recent metagenome studies revealed that microbial diversity and richness were higher in weaned piglets fed probiotics (21), and synbiotic combination of lactulose and probiotic enterococci (9). Our results support the evidence that effects of probiotics, prebiotics and synbiotics in microbial diversity varies widely in weaned pigs.

In addition, STEC challenge significantly decreased the alpha diversity in the non-treated CONT group, indicating low bacterial community diversity in weaned piglets without synbiotic treatment. In beef cattle operations, presence of pathogenic *E*. *coli* is correlated with lower bacterial community diversity and composition (22), since cattle are the most important STEC reservoir (23). However, no significant differences in alpha diversity were observed between piglets fed dietary treatments and STEC-challenged CONT group. This could explain that probiotics have protective effects against pathogenic bacteria by producing antimicrobial compounds, decreasing the intestinal pH, and competing with pathogens for adhesion and colonization in the gut (24, 25). Our results indicate that use of probiotics, prebiotics and their synbiotic combination is effective in reducing the negative effects of STEC and may balance and restore the gut microbial diversity in a piglet challenge model.

Beta-diversity analyses based on the PCoA plots using both unweighted and weighted UniFrac distances showed that microbial community structure was perturbed by oral administration of synbiotic additives and by challenge with STEC in weaned piglets. Both PCoA plots showed that microbiota of the non-challenged pigs fed additives were clustered separately, suggesting that administration of prebiotics, probiotics and synbiotics have unique effects on the microbial community structure in weaned piglets. However, oral challenge of STEC in treatment groups lead to segregated clustering of the microbiota, suggesting that STEC caused perturbation of the microbiota in weaned piglets receiving prebiotics, probiotics and synbiotics. To verify this result, microbiota of the non-challenged CONT group were compared to the STEC-challenged CONT, resulting to significant clustering of the two groups. This suggest that STEC significantly altered the microbial community structure and composition regardless of oral administration of prebiotics, probiotics or their synbiotic combination in weaned piglets. Our results were similar to those obtained by Chae et al. (9) who showed significant clustering of pig gut microbiota according to dietary treatment of prebiotics, probiotics and their synbiotic combination. Furthermore, findings of this study suggest that prebiotics, probiotics and synbiotics in addition to oral challenge of STEC in pigs had individual effects on intestinal microbial community structure in pigs. The results of the present study also indicate that STEC is associated with gut microbiota dysbiosis, while prebiotics, probiotics and synbiotics have inhibitory effects against STEC.

Consistent with a previous study on pig gut microbiota (26), Firmicutes and Bacteroidetes were the two most abundant taxa at the phylum level. Previously, an increased in Firmicutes to Bacteroidetes ratio was detected in piglets after oral administration of prebiotics, probiotics and synbiotics (9). While intestinal microbial communities play important roles in modulating host physiology (27), Firmicutes to Bacteroidetes ratio in pig gut microbiota is a major contributor to adiposity (28, 29). Firmicutes has key roles in starch and fiber degradation (30, 31) and Bacteroidetes contribute significantly in degradation of proteins and polysaccharides in the plant cell wall, producing short-chain fatty acids (SCFAs) that can be absorbed by the host (32) and they can modify the host lipid metabolism, increasing fat retention and adipogenesis (33). Conversely, the present study observed higher Firmicutes to Bacteroidetes ratio in CONT group than in the treatment groups, suggesting that supplementation of prebiotics, probiotics and synbiotics have varying effects on pig microbiota and may improve gut health and performance of piglets. In addition, we detected in this study that the phylum Tenericutes was significantly depleted by oral administration of prebiotics, probiotics and synbiotics. It has been reported that Tenericutes are correlated with apparent crude fiber digestibility in pigs (34) and they are associated with healthy human gut microbiota along with decreased abundance of Proteobacteria, which are known pathogenic phyla (35). These findings imply that synbiotic administration significantly improved the gut health status of weaned piglets by decreasing the populations of pathogenic bacteria. Interestingly, STEC challenge had no significant effects on microbial composition at the phylum level in pigs fed the dietary treatment. In a previous report, it has been suggested that synbiotics are as effective as antibiotics on growth performance, nutrient digestibility and enhancement of gut microbiota in weaned piglets (36). Our study suggests that prebiotics, probiotics, and synbiotics have protective effects against pathogenic STEC and induce beneficial effects in improving gut health in weaned piglets. The discrepancies between the present study and previous studies may have resulted from the use of pigs in different ages, environmental conditions, probiotic strains and types of prebiotics.

To further illuminate whether the changes in the composition of the microbiota were associated with dietary treatment and STEC infection, the distribution of microbiota at the genus level were investigated. At the genus level, *Prevotella* and *Lactobacillus* were detected as the two most abundant genera in the swine gut microbiota regardless of synbiotic supplementation and STEC challenge. Interestingly, PRO and SYN1 significantly elevated the relative abundance of *Lactobacillus*, while it decreased in PRE in comparison to the CONT group. It has been reported that *Lactobacillus* is the most common probiotic agent in swine production due to their ability to improve growth performance and prevent gastrointestinal infection (37). Moreover, *Lactobacillus* has been known to improve feed conversion efficiency, nutrient utilization, intestinal microbiota, gut health and regulation of immune function in pigs (38). Interestingly, STEC challenge significantly depleted the abundance of *Lactobacillus* in the negative CONT group, however PRO reversed this result, suggesting that probiotics may play a role in proliferation of beneficial organisms during infection and fight against pathogenic bacteria. In a similar study, synbiotic combination of *L*. *plantarum* with maltodextrins and fructooligosaccharides showed significant reductions in the population of *E*. *coli* K-88 in the intestinal mucosa in pigs (39). Moreover, in the present study, *Prevotella* was significantly elevated in PRE, PRO, SYN1 and SYN2 as compared to the non-challenged CONT. *Prevotella* strains are associated with plant-rich diets but are also linked with chronic inflammatory conditions (40). Previously, the abundance of *Prevotella* was significantly increased with supplementation of fermentable non-starch polysaccharides (41) and low-molecular-weight chitosan which are potential prebiotics in weaned piglets (42). One of the most striking observation in this study was the significant depletion of *Phascolarctobacterium* in all dietary treatment groups challenged with STEC. *Phascolarctobacterium* was found to be a beneficial microbe that play a significant role in SCFA production including acetate and propionate and can be associated with the metabolic state and mood of the host (43). Conclusively, it could be speculated that synbiotics improve the survival and activity of beneficial microorganisms mainly due to synergistic effects of prebiotic and probiotics in the regulation of intestinal microbiome.

## Conclusion

It is evident that the pig intestinal microbiome plays an important role in modulating gut health and disease. In this study, lactulose and *Pediococcus acidilactici* showed unique effects and their synbiotic combination resulted in different alterations of the gut microbial communities in weaned piglets. Oral supplementation of prebiotics, probiotics and their synbiotic combination modulated the pig gut microbiota by increasing the abundance of beneficial microbes including *Lactobacillus* and *Prevotella*. Conclusively, it could be speculated that synbiotics 1 (SYN1) was most effective on improving the activity of beneficial microorganisms and growth performance mainly due to synergistic effects of prebiotic and probiotics. Overall, findings of this study may be used for development feeding strategies such as alternatives to in-feed antibiotics in the swine production for intestinal development and modulation of the gut microbiota.

## Data availability statement

All raw 16S rRNA gene data used in this study were deposited in the National Center for Biotechnology Information (NCBI) under BioProject accession number PRJNA576307.

## Ethics statement

The animal study was reviewed and approved by Institutional Animal Care and Use Committee of the Dankook University, South Korea (Approval number: DK-1-1645).

## Author contributions

JiC, MS, SW, CL, WC, and HBK designed the research. RG, JaC, JL, HK, SK, EK, GK, and CL participated in animal trial and data analysis. RG, MS, and SK validated and interpreted the data. RG, JaC, MS, SW, and HBK wrote the manuscript. HBK were the principal investigator. All authors read and approved the final manuscript.
