# Supplementary material for: Gut microbial shifts by synbiotic combination of Pediococcus acidilactici and lactulose in weaned piglets challenged with Shiga toxin-producing Escherichia coli
Source: Front Vet Sci. 2023 Jan 12;9:1101869. doi: 10.3389/fvets.2022.1101869 (PMC9879705; doi:10.3389/fvets.2022.1101869)
Supplement: Supplementary file 1 [file Presentation_1.PPTX]

## Slide 1
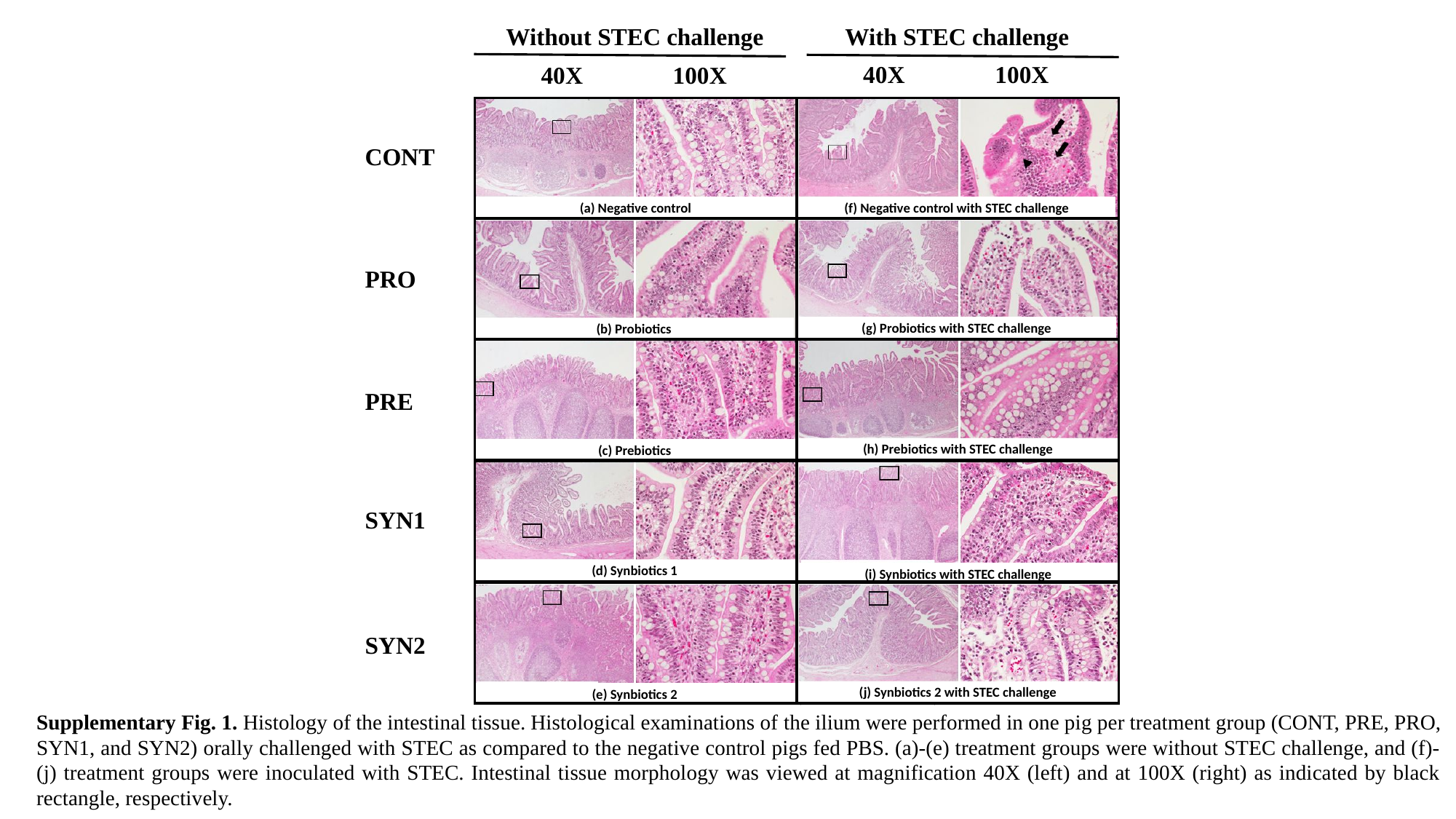

Without STEC challenge
With STEC challenge
40X 100X
40X 100X
CONT
(a) Negative control
(f) Negative control with STEC challenge
PRO
(g) Probiotics with STEC challenge
(b) Probiotics
PRE
(h) Prebiotics with STEC challenge
(c) Prebiotics
SYN1
(d) Synbiotics 1
(i) Synbiotics with STEC challenge
SYN2
(j) Synbiotics 2 with STEC challenge
(e) Synbiotics 2
Supplementary Fig. 1. Histology of the intestinal tissue. Histological examinations of the ilium were performed in one pig per treatment group (CONT, PRE, PRO, SYN1, and SYN2) orally challenged with STEC as compared to the negative control pigs fed PBS. (a)-(e) treatment groups were without STEC challenge, and (f)-(j) treatment groups were inoculated with STEC. Intestinal tissue morphology was viewed at magnification 40X (left) and at 100X (right) as indicated by black rectangle, respectively.

## Slide 2
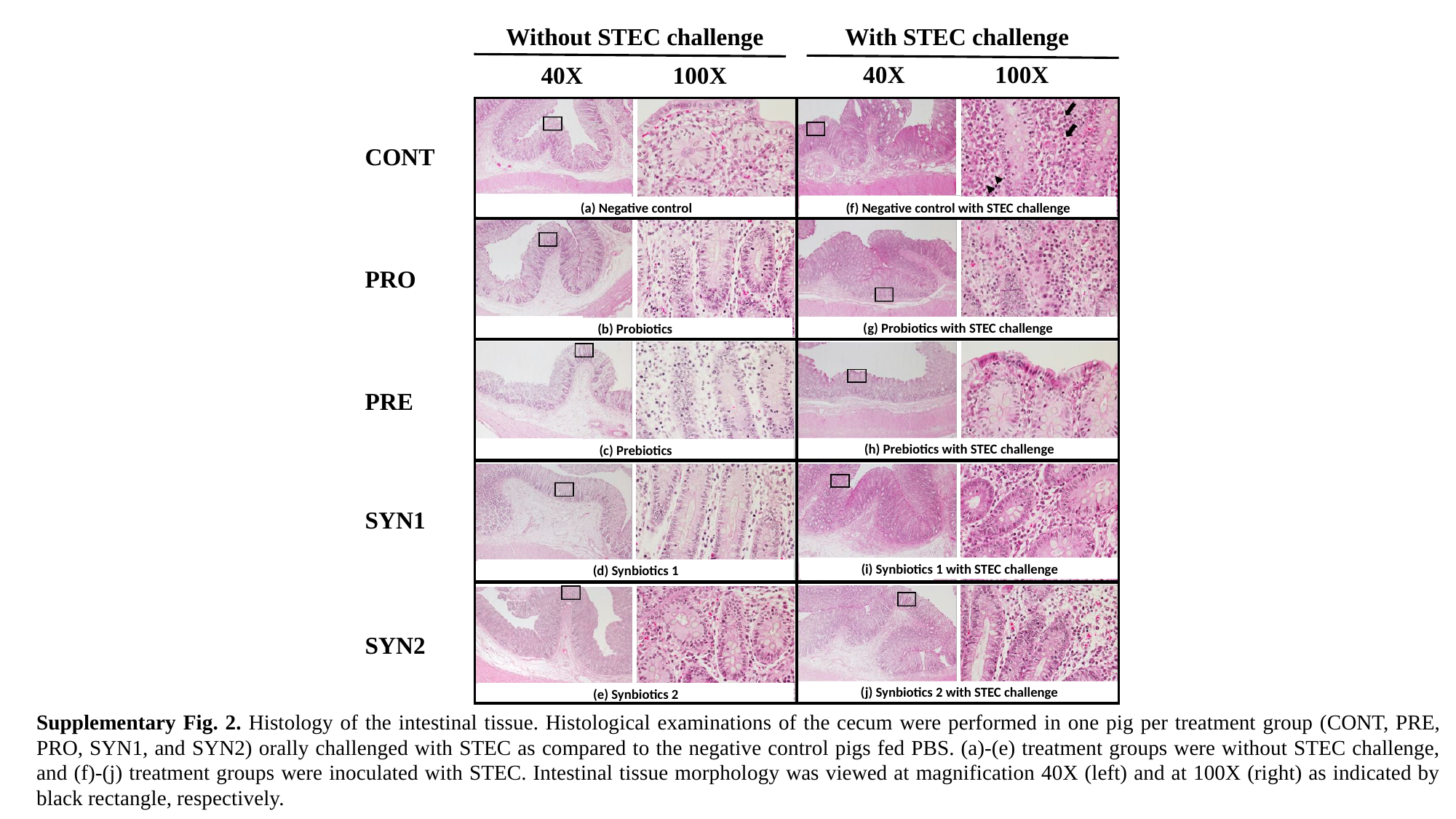

Without STEC challenge
With STEC challenge
40X 100X
40X 100X
CONT
(a) Negative control
(f) Negative control with STEC challenge
PRO
(g) Probiotics with STEC challenge
(b) Probiotics
PRE
(h) Prebiotics with STEC challenge
(c) Prebiotics
SYN1
(i) Synbiotics 1 with STEC challenge
(d) Synbiotics 1
SYN2
(j) Synbiotics 2 with STEC challenge
(e) Synbiotics 2
Supplementary Fig. 2. Histology of the intestinal tissue. Histological examinations of the cecum were performed in one pig per treatment group (CONT, PRE, PRO, SYN1, and SYN2) orally challenged with STEC as compared to the negative control pigs fed PBS. (a)-(e) treatment groups were without STEC challenge, and (f)-(j) treatment groups were inoculated with STEC. Intestinal tissue morphology was viewed at magnification 40X (left) and at 100X (right) as indicated by black rectangle, respectively.

## Slide 3
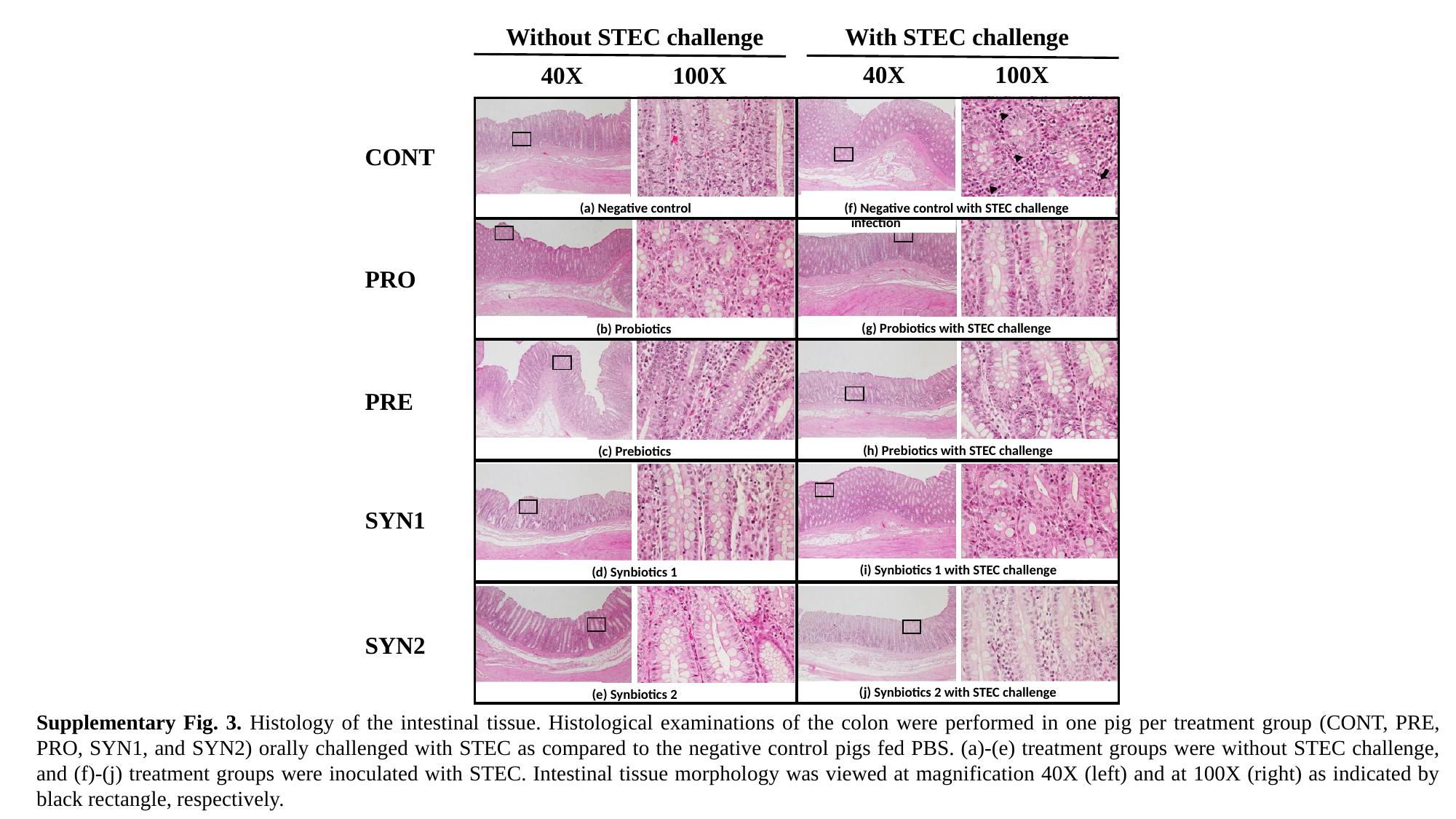

Without STEC challenge
With STEC challenge
40X 100X
40X 100X
CONT
(f) Negative control- with infection
(a) Negative control
(f) Negative control with STEC challenge
PRO
(g) Probiotics with STEC challenge
(b) Probiotics
PRE
(h) Prebiotics with STEC challenge
(c) Prebiotics
SYN1
(i) Synbiotics 1 with STEC challenge
(d) Synbiotics 1
SYN2
(j) Synbiotics 2 with STEC challenge
(e) Synbiotics 2
Supplementary Fig. 3. Histology of the intestinal tissue. Histological examinations of the colon were performed in one pig per treatment group (CONT, PRE, PRO, SYN1, and SYN2) orally challenged with STEC as compared to the negative control pigs fed PBS. (a)-(e) treatment groups were without STEC challenge, and (f)-(j) treatment groups were inoculated with STEC. Intestinal tissue morphology was viewed at magnification 40X (left) and at 100X (right) as indicated by black rectangle, respectively.

## Slide 4
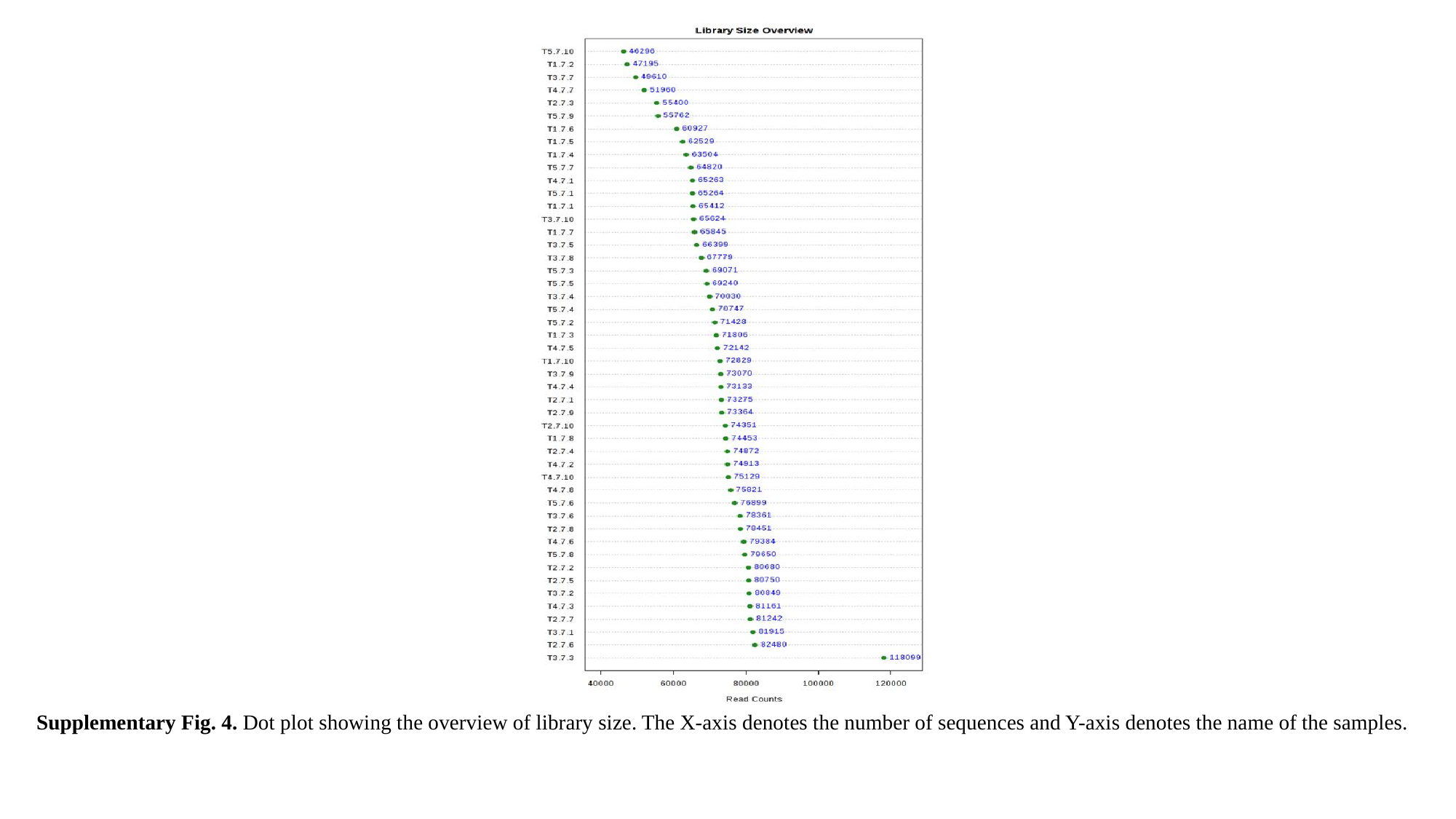

Supplementary Fig. 4. Dot plot showing the overview of library size. The X-axis denotes the number of sequences and Y-axis denotes the name of the samples.

## Slide 5
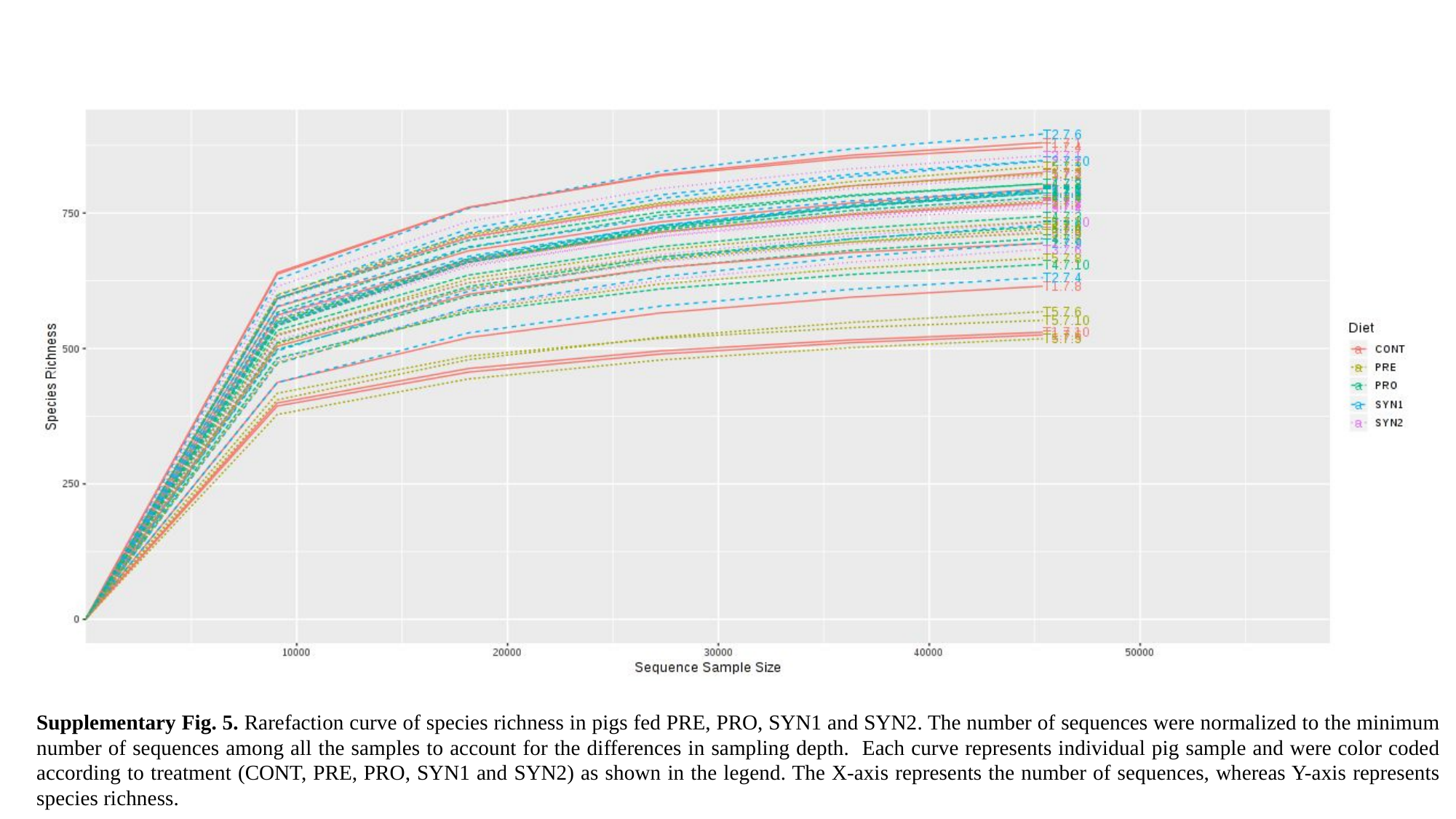

Supplementary Fig. 5. Rarefaction curve of species richness in pigs fed PRE, PRO, SYN1 and SYN2. The number of sequences were normalized to the minimum number of sequences among all the samples to account for the differences in sampling depth. Each curve represents individual pig sample and were color coded according to treatment (CONT, PRE, PRO, SYN1 and SYN2) as shown in the legend. The X-axis represents the number of sequences, whereas Y-axis represents species richness.

## Slide 6
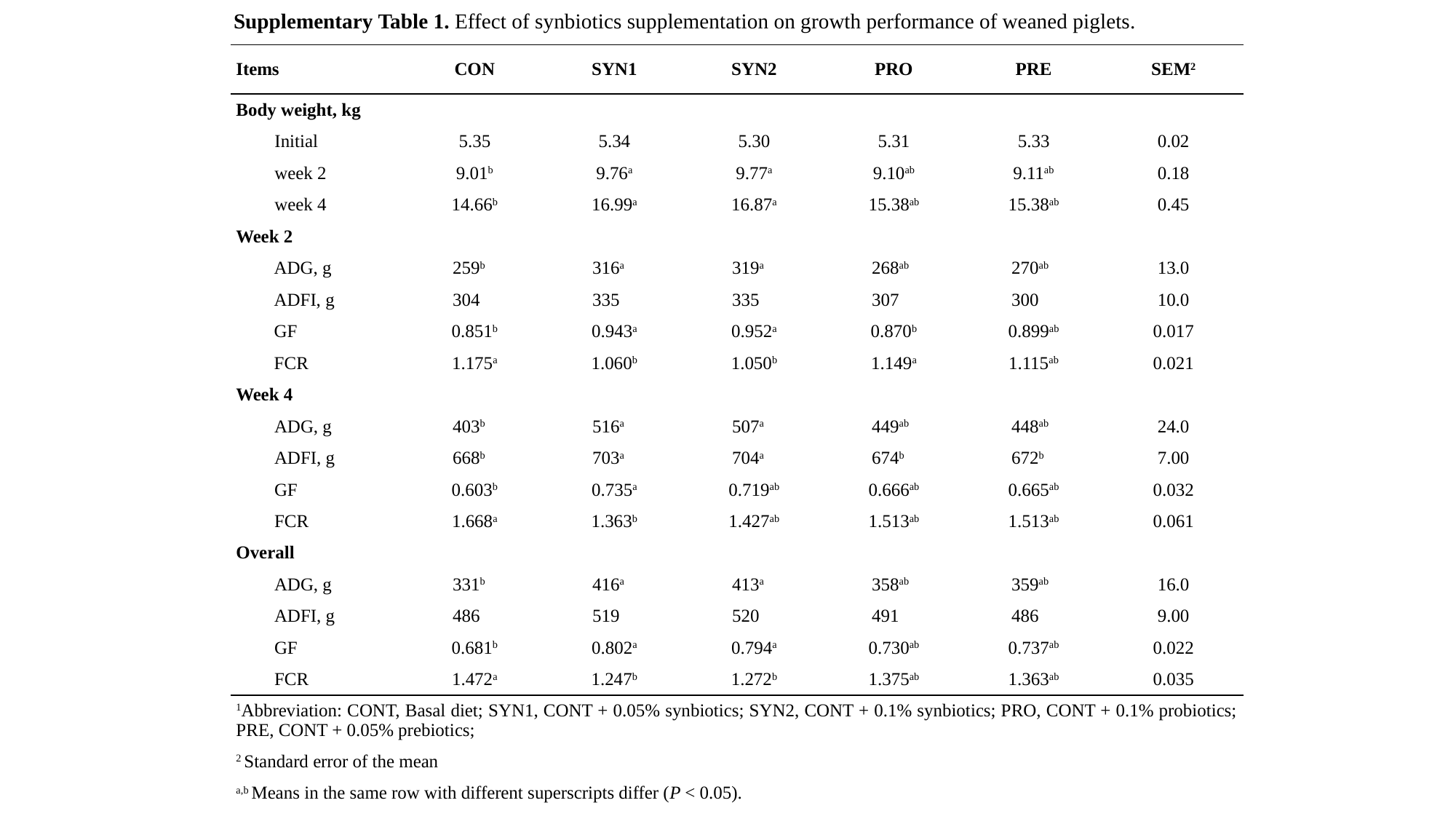

Supplementary Table 1. Effect of synbiotics supplementation on growth performance of weaned piglets.
| Items | CON | SYN1 | SYN2 | PRO | PRE | SEM2 |
| --- | --- | --- | --- | --- | --- | --- |
| Body weight, kg | | | | | | |
| Initial | 5.35 | 5.34 | 5.30 | 5.31 | 5.33 | 0.02 |
| week 2 | 9.01b | 9.76a | 9.77a | 9.10ab | 9.11ab | 0.18 |
| week 4 | 14.66b | 16.99a | 16.87a | 15.38ab | 15.38ab | 0.45 |
| Week 2 | | | | | | |
| ADG, g | 259b | 316a | 319a | 268ab | 270ab | 13.0 |
| ADFI, g | 304 | 335 | 335 | 307 | 300 | 10.0 |
| GF | 0.851b | 0.943a | 0.952a | 0.870b | 0.899ab | 0.017 |
| FCR | 1.175a | 1.060b | 1.050b | 1.149a | 1.115ab | 0.021 |
| Week 4 | | | | | | |
| ADG, g | 403b | 516a | 507a | 449ab | 448ab | 24.0 |
| ADFI, g | 668b | 703a | 704a | 674b | 672b | 7.00 |
| GF | 0.603b | 0.735a | 0.719ab | 0.666ab | 0.665ab | 0.032 |
| FCR | 1.668a | 1.363b | 1.427ab | 1.513ab | 1.513ab | 0.061 |
| Overall | | | | | | |
| ADG, g | 331b | 416a | 413a | 358ab | 359ab | 16.0 |
| ADFI, g | 486 | 519 | 520 | 491 | 486 | 9.00 |
| GF | 0.681b | 0.802a | 0.794a | 0.730ab | 0.737ab | 0.022 |
| FCR | 1.472a | 1.247b | 1.272b | 1.375ab | 1.363ab | 0.035 |
| 1Abbreviation: CONT, Basal diet; SYN1, CONT + 0.05% synbiotics; SYN2, CONT + 0.1% synbiotics; PRO, CONT + 0.1% probiotics; PRE, CONT + 0.05% prebiotics; | | | | | | |
| 2 Standard error of the mean | | | | | | |
| a,b Means in the same row with different superscripts differ (P < 0.05). | | | | | | |

## Slide 7
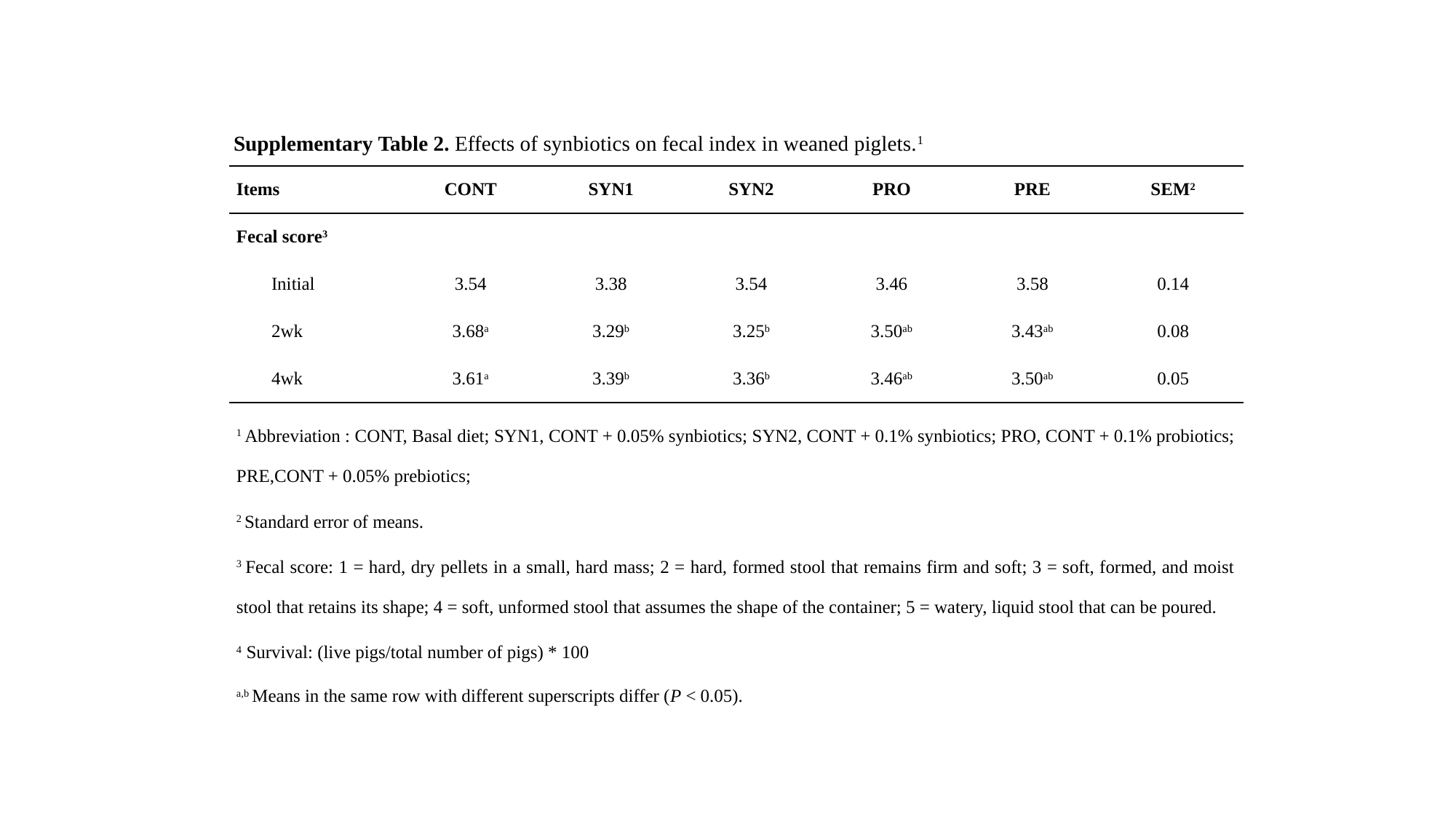

Supplementary Table 2. Effects of synbiotics on fecal index in weaned piglets.1
| Items | CONT | SYN1 | SYN2 | PRO | PRE | SEM2 |
| --- | --- | --- | --- | --- | --- | --- |
| Fecal score3 | | | | | | |
| Initial | 3.54 | 3.38 | 3.54 | 3.46 | 3.58 | 0.14 |
| 2wk | 3.68a | 3.29b | 3.25b | 3.50ab | 3.43ab | 0.08 |
| 4wk | 3.61a | 3.39b | 3.36b | 3.46ab | 3.50ab | 0.05 |
| 1 Abbreviation : CONT, Basal diet; SYN1, CONT + 0.05% synbiotics; SYN2, CONT + 0.1% synbiotics; PRO, CONT + 0.1% probiotics; PRE,CONT + 0.05% prebiotics; | | | | | | |
| 2 Standard error of means. | | | | | | |
| 3 Fecal score: 1 = hard, dry pellets in a small, hard mass; 2 = hard, formed stool that remains firm and soft; 3 = soft, formed, and moist stool that retains its shape; 4 = soft, unformed stool that assumes the shape of the container; 5 = watery, liquid stool that can be poured. | | | | | | |
| 4 Survival: (live pigs/total number of pigs) \* 100 | | | | | | |
| a,b Means in the same row with different superscripts differ (P < 0.05). | | | | | | |
